# The impact of *i*-PUSH on maternal and child health care utilization, health outcomes, and financial protection: study protocol for a cluster randomized controlled trial based on financial and health diaries data

**DOI:** 10.1186/s13063-021-05598-7

**Published:** 2021-09-15

**Authors:** Amanuel Abajobir, Richard de Groot, Caroline Wainaina, Anne Njeri, Daniel Maina, Silvia Njoki, Nelson Mbaya, Hermann Pythagore Pierre Donfouet, Menno Pradhan, Wendy Janssens, Estelle M. Sidze

**Affiliations:** 1 African Population and Health Research Center, Nairobi, Kenya; 2 Amsterdam Institute of Global Health and Development, Amsterdam, Netherlands; 3 Vrije Universiteit Amsterdam, Amsterdam, Netherlands

**Keywords:** Maternal and child health, Health care utilization, Out-of-pocket health expenditures, Health insurance, Universal Health Coverage, Cluster randomized controlled trial, Digital tools, Mobile health, Kenya

## Abstract

**Background:**

Universal Health Coverage ensures access to quality health services for all, with no financial hardship when accessing the needed services. Nevertheless, access to quality health services is marred by substantial resource shortages creating service delivery gaps in low-and middle-income countries, including Kenya. The Innovative Partnership for Universal Sustainable Healthcare (*i*-PUSH) program, developed by AMREF Health Africa and PharmAccess Foundation (PAF), aims to empower low-income women of reproductive age and their families through innovative digital tools. This study aims to evaluate the impact of *i*-PUSH on maternal and child health care utilization, women’s health including their knowledge, behavior, and uptake of respective services, as well as women’s empowerment and financial protection. It also aims to evaluate the impact of the LEAP training tool on empowering and enhancing community health volunteers’ health literacy and to evaluate the impact of the M-TIBA health wallet on savings for health and health insurance uptake.

**Methods:**

This is a study protocol for a cluster randomized controlled trial (RCT) study that uses a four-pronged approach—including year-long weekly financial and health diaries interviews, baseline and endline surveys, a qualitative study, and behavioral lab-in-the-field experiments—in Kakemega County, Kenya. In total, 240 households from 24 villages in Kakamega will be followed to capture their health, health knowledge, health-seeking behavior, health expenditures, and enrolment in health insurance over time. Half of the households live in villages randomly assigned to the treatment group where *i*-PUSH will be implemented after the baseline, while the other half of the households live in control village where *i*-PUSH will not be implemented until after the endline. The study protocol was reviewed and approved by the AMREF Ethical and Scientific Review Board. Research permits were obtained from the National Commission for Science, Technology and Innovation agency of Kenya.

**Discussion:**

People in low-and middle-income countries often suffer from high out-of-pocket healthcare expenditures, which, in turn, impede access to quality health services. Saving for healthcare as well as enrolment in health insurance can improve access to healthcare by building capacities at all levels—individuals, families, and communities. Notably, *i*-PUSH fosters savings for health care through the mobile-phone based “health wallet,” it enhances enrolment in subsidized health insurance through the mobile platform—M-TIBA—developed by PAF, and it seeks to improve health knowledge and behavior through community health volunteers (CHVs) who are trained using the LEAP tool—AMREF’s mHealth platform. The findings will inform stakeholders to formulate better strategies to ensure access to Universal Health Coverage in general, and for a highly vulnerable segment of the population in particular, including low-income mothers and their children.

**Trial registration:**

Registered with Protocol Registration and Results System (protocol ID: AfricanPHRC; trial ID: NCT04068571: AEARCTR-0006089; date: 29 August 2019) and The American Economic Association’s registry for randomized controlled trials (trial ID: AEARCTR-0006089; date: 26 June 2020).

## Background

There has been a renewed international commitment to Universal Health Coverage (UHC) aiming at ensuring all people have access to the health services they need without suffering from financial hardships. However, there are sustained resource shortages and service delivery gaps in many countries that prevent them from meeting the Sustainable Development Goal (SDG) 3.8 related to UHC (i.e., achieve UHC, including financial risk protection, access to quality essential health care services and access to safe, effective, quality, and affordable essential medicines and vaccines for all). Achieving UHC is particularly important in achieving SDGs related to maternal and child health (i.e., reducing the global maternal mortality ratio to less than 70 per 100 000 live births (SDG 3.1) and ending preventable deaths of newborns and children under 5 years of age (SDG 3.2). All countries are committed to reduce neonatal mortality to at least as low as 12 per 1000 live births and under-5 mortality to at least as low as 25 per 1000 live births by 2030. Evidence still shows that more than half of the world’s population lacks access to health care of sufficient quality [1] and about 100 million people fall into extreme poverty each year due to ill-health [2], particularly in low- and middle-income countries (LMICs). Systemic reforms are needed to translate commitment to UHC into a reality.

Despite being classified as a middle-income country in 2014, Kenya still remains among the 25% poorest countries strongly affected by social and health inequalities in the world. More than one third of Kenyans have an income below the poverty line (1.9 USD/day) [1–5]. Inequalities in access to healthcare, in particular maternal and child health care, are still rampant, despite major improvements made through targeted policies over the past few years [5]. For instance, according to the 2014 Kenya Demographic and Health Survey (KDHS), the maternal mortality ratio has marginally reduced to 362 per 100,000 live births, not statistically different from the figures reported in 2008–2009 [4]. While a substantial under-5 mortality reduction was achieved with a drop from 115 per 1000 live births in 2003 to 52 per 1000 live births in 2014, it is still twofold higher than the SDG target. These figures are disproportionately higher in Western Kenya, such as in Kakamega County. The poorest mothers are still far behind in terms of coverage of essential Reproductive and Maternal and Child Health services [4, 5].

However, there is little evidence on the effectiveness of intervention strategies in enhancing the coverage, integration, and implementation of maternal and child health services in primary healthcare system in developing countries, including Kenya. Interestingly, the Government of Kenya has included UHC as one of its “Big Four Agenda”-action points, which is anticipated to lead the transformation of the country by 2022 [3, 4]. The objective is to achieve a 100% cost subsidy for essential health services and to reduce out-of-pocket health expenditures by half. Low-cost health insurance schemes, eHealth, and mobile health (mHealth) services are among other opportunities to achieve this goal. Consequently, two non-governmental organizations, AMREF Health Africa and PharmAccess Foundation (PAF), are both supporting the Government to achieve the goal of UHC in many counties. This includes Kakamega—a county in Western Kenya—where AMREF and PAF are jointly implementing their Innovative Partnership for Universal Sustainable Healthcare (*i*-PUSH) program.

In order to maximize the effectiveness of this program, it is important for stakeholders to have a deep understanding of current access to health services, households’ health-related decision-making, and out-of-pocket health care expenditures in the target populations. The main aim of the study is to evaluate the impact of *i*-PUSH (most notably enhanced access to subsidized health insurance through the “health wallet” and increased community health volunteer (CHV) training opportunities through the LEAP training tool) on health care utilization, in particular related to maternal and child health, and financing of out-of-pocket health care expenditures. The evaluation research is conducted by two independent research institutes, namely, the African Population and Health Research Center (APHRC) and Amsterdam Institute for Global Health and Development (AIGHD). It is funded by the Dutch Postal Code Lottery, the Joep Lange Institute, and the Dutch Ministry of Foreign Affairs through the Health Insurance Fund.

## Methods/design

### Study site and period

*i*-PUSH has been ongoing in parts of Kakamega County since 2017. The study is being carried out in Khwisero Sub-county—one of the sub-counties in Kakamega County, where the *i*-PUSH program has expanded after the baseline survey. The implementing partners selected two health clinics that were considered to be included in the expansion of the *i*-PUSH program. Twenty-four (24) villages located in the catchment areas of these two clinics were randomly selected from a list of all eligible villages (*N* = 239) in the catchment areas. The list of villages was provided by the Sub-county government jointly with the *i*-PUSH program area manager. Random selection was done by the research team using a computer program to generate a short list of villages from the longlist, to be randomly assigned to either the treatment or the control group. Villages cover on average about 100 households. Each village is served by one unique CHV.

### Study design and randomization procedure

The survey design is a longitudinal cluster randomized controlled trial (RCT). Randomization occurs at the level of villages in Khwisero Sub-county in Kenya. The “treatment” and “control” groups are constructed, comparing villages where *i*-PUSH has been rolled out after the baseline with villages where *i*-PUSH would not roll out until after the endline. The research team used community-level socio-demographic and infrastructure indicators (including number of households, village-level access to basic amenities and public services, adult literacy rate, women literacy rate, perceived health status, and healthcare utilization indicators) from baseline data to form pairs of similar villages and determine the exact matching indicators.

In keeping with robustness of the cluster RCT, the procedure hence followed four steps for matching of the “treatment” and “control” villages: (i) purposive selection of the Sub-county (Khwisero) where the intervention rolled out, (ii) random selection of 24 villages, (iii) pair-matching of villages based on relevant background characteristics and baseline outcomes of interest, and (iv) randomization of treatment and control villages within each pair of villages by flipping a coin. Pairing villages before randomization reduces the risk of a bad draw during the randomization process. Randomization without pairing would, in expectation, also lead to similar control and treatment groups, but it was also possible that the random draw produces a control and treatment group with very different characteristics by chance [6]. This risk was reduced through pairing. We used the Euclidean distance for our matching process, which corresponds to the absolute difference between the standardized values of all of the covariates for a possible pair of matches. We conducted the matching within each of the four originally selected health clinic catchment areas separately. Thus, each village was matched with one of the other five villages in the vicinity of the health clinic. This was done to ensure that each health clinic had an equal number of treatment and control villages in their catchment area. Hence, we computed this distance measure between each village and all other villages within the same health clinic catchment area; “pair” the two villages with the minimum distance and remove them from the list; repeat the distance calculation excluding the pair made; and continue until all villages were paired.

After the matching process, the randomization assignment was carried out in the presence of key stakeholders including PAF, local liaison persons and village representatives, upon explaining all steps. Consent for the procedures was obtained from local government officials before the random assignment. The following steps were followed: papers with paired village names were folded and put in a bag; and two village representatives from each paired village discussed on whom to pick the paper and after the other group members verified that the names could not be seen, one paper was picked. A Kenya Shilling 10 coin was used to decide which group the picked village belonged to by flipping the coin. The village representatives had decided that the head of the coin should represent the control group, justifying that Kenyatta (1st president of Kenya) was a “controlling village” and the shield to represent the intervention group. The process of choosing the folded paper and flipping of the coin was repeated for all paired villages.

The treatment group thus consists of the target population living in the randomly assigned 12 treatment villages. *i*-PUSH roll out in the treatment villages includes training of their CHVs with the LEAP tool, who subsequently introduced the health wallet to eligible women living in the treatment villages they serve, and offer them the subsidized insurance scheme on their mobile phone. The CHVs working in the control villages (as well as the remaining non-sampled villages on the longlist) have not received training on the LEAP tool until endline, nor were women in the control villages offered the health wallet and subsidized insurance on their mobile phone.

We randomized at the village level because (1) villages are served by one CHV each, who are either trained or not trained on LEAP (hence, the LEAP intervention cannot be varied within villages); (2) to avoid contamination between households within the same villages regarding health-related knowledge and behavior; and, (3) moreover, it was deemed politically unfeasible to offer the health wallet and subsidized health insurance to some eligible households in a village but not to other eligible households in that same village. Upon roll out of the subsidized health services, eligible households were encouraged to use the services, though they were given the right to opt out at any time.

### Study population

The study population consists of eligible households living in the selected study villages. Eligible households included those with at least one woman of reproductive age (WRA) (18–49) who (a) had at least one child below 4 years living with her at baseline or (b) was pregnant at baseline. Data on all household members are also collected from these eligible households.

Selected CHVs and PAF’s area manager provided the full list of households and other necessary information within the work area of each CHV. Based on the household demographics and pregnancy information, eligible households were identified. Initially, the study sought a 50-50 allocation between households with a pregnant woman and households with a child under 4 years old. After the household listing exercise, it became clear that there were too few pregnant women in each village to fulfill this criterion. We then decided to include all pregnant women in our sample and randomly sample additional households with children under 4 years old until the cluster size (10 households per village) was achieved. The research team did a random selection as follows: all eligible households with children under 4 years old were entered in a spreadsheet and receive a randomly assigned number. The team ordered the households per CHV based on this random number, and the first 10 households per CHV in each village were included in the study sample. Additional eligible households per CHV were over-sampled to serve as replacement households for dropouts.

### Sample size

Sample size calculation followed Hemming et al. [7]’s study by fixing the number of clusters per arm to be 12 clusters, and then estimated the cluster size and total sample size. In the current study, it was assumed that the *i*-PUSH program could yield an effect size of 0.4 standard deviation in terms of health care utilization with an intracluster correlation (ICC) of *ρ* = 0.014. The estimates of the ICC were derived from Geng et al. [8]’s, study conducted in Nandi County which used high-frequency data on diaries on health-seeking behaviors and financial expenditures over 1 year (October 2012–October 2013). The calculation of the ICC was based on health care utilization measured as visits to any formal health provider, unconditional on reported health symptoms. It hypothesized a confidence interval of 95%, a margin-of-error of 5%, and a power of 80%. With a cluster size per arm of 12 clusters, and a total number of women per cluster of 10, the total sample size was 120 households per arm and 240 households for the full study.

To keep sample size at par, households that dropped out of the study before the start of the intervention were replaced with new eligible households on a rolling basis for a maximum period of six months, or until the program started.

### Description of the *i-*PUSH program

*i*-PUSH is a comprehensive intervention that ultimately aims to improve the utilization of Reproductive and Maternal and Child Health (RMNCH) services among WRA and their young children in Kakamega and Nairobi Counties, by increasing knowledge about and (financial) access to RMNCH services as well as improving the quality of care of RMNCH services. In the original *i*-PUSH program that is the focus of this evaluation, households receive the first year of health insurance premium for free, while they are stimulated and supported to save for a 50% co-payment in the second year, and a 100% premium payment thereafter. The free provision in year one is expected to show the benefits of insurance to the selected households. The support for savings during the first year for a 50% co-payment in the second year is expected to install a habit of savings.^1^

The *i*-PUSH program utilizes innovative digital tools developed by both partners to enhance access to affordable and quality health care to low-income WRA and their families. Through *i*-PUSH, selected clinics participate in the “SafeCare” quality improvement program. Women receive the National Health Insurance Fund (NHIF) *SupaCover* at subsidized premiums on their mobile phone, using PAF’s so-called “health wallet.” The “health wallet” runs on the digital platform M-TIBA, which registers health care utilization at participating clinics, connecting patients, providers, and payers on one platform. PAF is piloting several other tools on this platform in addition to the health wallet, such as Connected Diagnostics for Malaria in Kisumu County, the MomCare program for antenatal and postnatal care in Kisumu and Nairobi Counties, and socio-economic mapping for UHC support in Kisumu County. CHVs are the first point-of-contact for women in the program. They make use of AMREF’s *Mjali* (Mobile Jamii Afya Link) tool for digital registration of household information and the mobile phone-based LEAP tool for training. The LEAP tool employs a mobile learning approach to train and empower CHVs using their mobile devices operating from any phone [9]. This enables the CHVs to learn at their own pace, and with their own mobile devices while in the community, providing both interpersonal and community aspects of learning. This evaluation study will focus on two of these spheres of interest supported by mobile tools: knowledge on health and health behavior and (financial) access to healthcare services; the quality upgrades at the health facilities cannot be evaluated with our study design because all *i*-PUSH clinics in our study area were already upgraded at baseline. A rough sketch to the implementation of enrolment, intervention, and assessment of the program is indicated in Table 1. The implementation of *i-*PUSH will not alter access to the usual health care services (including use of any medication) throughout the implementation of the program.

**Table 1:**
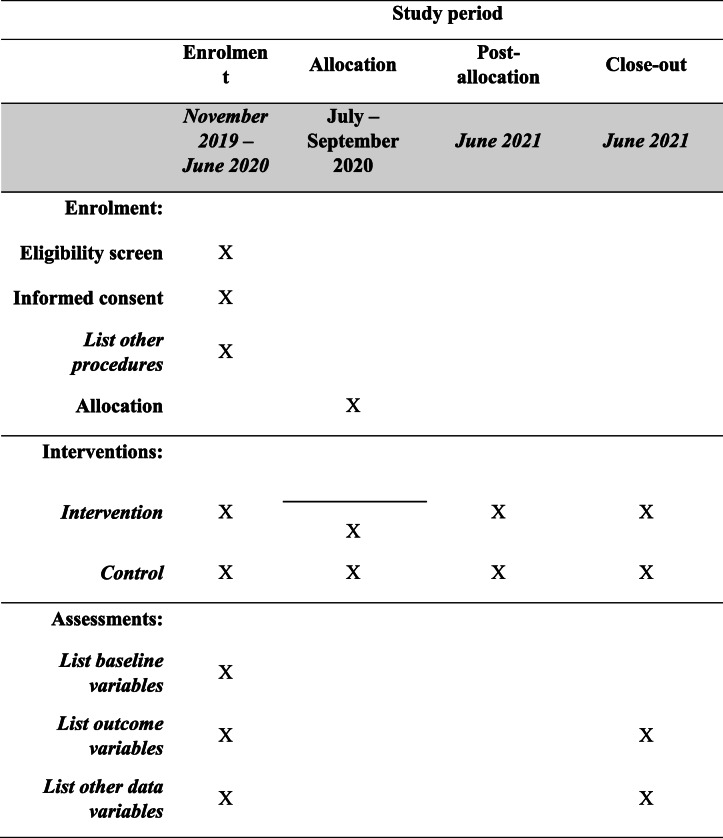
Schedule of enrolment, interventions, and assessments

### The LEAP training tool for CHVs

Working with CHVs, *i*-PUSH will increase the knowledge on both RMNCH and on insurance/health financing among women and men in the treatment communities. To this end, CHVs receive additional training through the specially developed LEAP training tool (module) carried out by AMREF. This tool contains modules on specific health terrains of interest (notably, health promotion activities for children under 5, family planning, antenatal care (ANC), danger signs in pregnancy and after delivery, danger signs in children under 5, maternal and child health and nutrition, water safety, hygiene and sanitation) as well as on health savings and health insurance. The CHVs can follow this training on the smartphone that they will receive as part of their inclusion in the program. It complements the standard monthly training sessions of CHVs by AMREF.

AMREF can currently assess whether LEAP improves the knowledge of its CHVs because at the end of each training module, CHVs can participate in a quiz on the tool that tests their newly gained knowledge. We will examine whether the LEAP training tool on top of the standard training activities of CHVs translate into improving women and men’s knowledge and behavior on pre-specified topics. We will also assess whether CHVs’ time spent on LEAP, number of training modules completed, and scores on the LEAP quizzes predict impact on women and men in the communities.

### Subsidized access to NHIF SupaCover health insurance through the M-TIBA health wallet

The improved knowledge on health and health financing of women and men is also expected to translate into improved attitudes towards insurance and saving for health and insurance. To support these changes in knowledge and attitudes, WRA would receive the first year of their NHIF insurance premium at 100% subsidy and the second year at a 50% subsidy. The subsidies are expected to enhance initial enrolment in NHIF such that enrollees can experience first-hand the benefits of insurance. Moreover, this will allow women to be acquainted with regular savings for the insurance premium for the next year, such that they will get into the habit of recurrent savings and increasingly be able to frequently set aside small amounts of money.

An integral component of the *i*-PUSH program is the so-called “health wallet” on M-TIBA (a mobile payment platform). Most WRA in the target population have limited access to formal financial services such as bank savings accounts. The widespread availability of M-PESA opens a new avenue of change in this respect. To alleviate this constraint, *i*-PUSH offers women the opportunity to set aside money in a commitment savings device on their mobile phone. The only requirement is that the mobile number is registered on their personal name. This will allow them (and other people such as spouses, relatives) to transfer funds into the wallet through M-PESA, which are subsequently “reserved” for direct payment of medical costs at M-TIBA-connected health providers or for future payment of the annual insurance co-premium. This feature of the health wallet is expected to support women in their financial planning—funds transferred into the wallet are kept safe and secure until they are needed for health-related purposes. Additional small-scale interventions are added to the program to enhance further savings, such as the provision of a savings calendar. Thus, this component of the program expects to increase savings for health as well as uptake and renewal of health insurance among the target population.

Enrolment in health insurance is further facilitated through an additional feature of *i*-PUSH—CHVs can digitally enroll WRA and their household members (spouses, children, and other dependents) on the NHIF *SupaCover* as long as they have an ID or birth certificate available at the time of the CHV visit. The CHV uploads the required documents and takes care of the registration process on his or her smartphone (as provided by the *i*-PUSH program), saving the women from a lot of hassle in traveling back and forth to the insurance offices to hand over all the required documents and go through the administrative steps. In other words, *i*-PUSH is also expected to relieve logistical and time constraints to the uptake of insurance. Such a scheme enhances enrolment into insurance [9].

## Expected outcomes

Overall, the outcomes of interest for this evaluation study that are measured during the 18 months of follow-up include the following:

### Primary outcomes (health care utilization and health expenditures)

- Absolute number of visits to any formal health care provider for treatment of illness or injury (to measure curative healthcare utilization), over the full period between baseline and endline.

### Secondary outcomes

#### Health outcomes

- Proportion of children under 5 who are fully immunized (to measure preventive healthcare utilization among young children).
- Proportion of pregnant women who complete their maternal care journey, i.e., 4 ANC visits, skilled birth attendant, 1 PNC visit (to measure maternal healthcare utilization among pregnant women).
- Proportion of children malnourished (anthropometrics) for children under 5.
- Proportion of children under 5 experiencing common childhood illnesses (diarrhea, acute febrile illnesses, acute respiratory infections) for children under 5.
- Out-of-pocket health expenditures.

#### Health knowledge and behavior

- Knowledge on maternal and child health.
- Proportion of children under 5 sleeping under a bed net.

#### Women’s empowerment

- Financial and health related decision-making power of women.

## Data collection instruments and techniques

Data collection consists of four main components: (1) a qualitative study, (2) baseline and endline household surveys, (3) weekly financial and health diaries interviews with all adults and emancipated minors in the households, and (4) behavioral lab-in-the-field experiments. Both quantitative and qualitative tools were piloted in Nairobi slums and debriefed to the research team including the field team.

### Qualitative study

The study uses qualitative data collection methods to get a deeper understanding of the perceptions and behaviors of the population on health insurance and health care utilization, to feed into the quantitative instrument design at baseline and to complement findings from the quantitative surveys at endline. This will help understand the motivations, drivers, and obstacles to savings for insurance in the target population. The qualitative baseline study is based on in-depth interviews (IDIs, *n* = 20) and focus group discussions (FGD, *n* = 4) with eligible men and women who were purposively sampled after CHVs’ mobilization to willingly participate in the study. Another qualitative study will be conducted parallel to the quantitative endline survey to provide additional under-the-skin description and/or for improved interpretation if the impact evaluation yields unexpected results.

### Baseline and endline survey instruments

The quantitative evaluation starts with a baseline survey before the rollout of the *i*-PUSH program and is completed by an endline survey conducted approximately 1 year after the *i*-PUSH introduction. Both surveys include modules on household demographics, socio-economic indicators, food and non-food consumption indicators, financial inclusion, participation in community networks, as well as self-assessed health status, health-related knowledge and behavior, health care utilization and health expenditures, maternal health, mental health, intra-household decision-making processes, and gender dynamics. In addition, the endline survey will include a satisfaction module on participation in the *i*-PUSH program for women in the treatment areas only and will be collected virtually using telephone interviews due to the COVID-19 pandemic. The household head or the most knowledgeable household member responds to the household roster, household-level modules including health care utilization and expenditures, and questions about under-aged household members. The focal person for the modules on maternal health, intra-household decision-making and health knowledge and behavior is the WRA in the household. All remaining individual-level modules (mental health, preferred clinic, mobile money use, savings groups and financial instruments) are reported by all adult individuals in the household (Table 2 in Appendix).

### Financial and health diaries

This study uses weekly financial and health diaries data collection methodology as its core research instrument, because this technique is well suited to address the research objectives by providing a granular insight into the financial lives and health-related decisions of participating households. The diaries are collected over the full study period between baseline and endline survey. The financial diaries record all financial transactions such as purchases, gifts, remittances, and loans, including those between household members in the 7 days prior to each interview. Transactions are described by type, incoming or outgoing cash, amount, purpose, transaction partner, and date. The health diaries provide a detailed picture of the incidence of illnesses and injuries, as well as preventive and curative health-seeking behavior. Health diaries collect data on all health events that occurred to any of the household members (respondents, their children and other household members) in the 7 days prior to each interview. This includes symptoms, whether any health care was sought, which health provider was visited, health services received, out-of-pocket health expenditures, date of onset of the symptoms, and date of provider visit(s).

Respondents to the weekly diaries interviews are all adults in the study households who are economically active (i.e., who handle money, excluding young adults who are still studying) and capable of conducting the interview (i.e., excluding very old-age or disabled household members). Emancipated minors, i.e., teenage girls who are a mother or who are pregnant, are also included as diaries respondent. The financial diaries are reported by each individual him- or herself. The health diaries can be reported by one individual for the entire household.

Data are collected through structured and formatted digital tools that allow to analyze data as they are recorded and improve data collection tools mid-course of the process. The short recall period drastically reduces recall bias and ensures that both major and minor illness episodes, including those with foregone care are reported. Because interviewers visit households weekly, they build a relationship of trust, which enables the diaries to capture also more sensitive health events. This is of particular importance in relation to capturing maternal and child health experiences.

Qualified fieldworkers were recruited and trained on data collection tools and techniques. Diaries data are collected through personal interviews in a conversation-like manner. Interviewers record information navigating through a specialized software program while the interview unfolds. The tool was administered face-to-face using tablets and took about 10–15 min. Prior experience of the research team with similar data collection indicates that such interviews generally last 10–15 min per household as both interviewers and respondents get more experienced. To reduce burden on the respondents, interviewers take care in planning the day, time, and location of the weekly interviews at the convenience of the respondent. The respondents are interviewed separately and in private spaces to ensure confidentiality. The COVID-19 situation has switched the mode of data collection to telephone interviews. Fieldworkers were retrained on telephone interview ethics and techniques. This will be implemented until the crisis recedes.

At regular intervals, the weekly diaries are complemented with pop-up modules. In particular, these include on a monthly basis, a pregnancy module, perinatal depression, and general mental health, and on a quarterly basis, food consumption of children, anthropometrics of children and their mothers. Since the COVID-19 outbreak, a monthly module has been added to assess the effect of COVID-19 pandemic on COVID19-related knowledge, preventive behavior (such as hand-washing and social distancing), mobility, barriers to health-seeking behavior, and fear/worries.

### Behavioral lab-in-the-field experiments

Diaries respondents are also invited to participate in a number of incentivized behavioral lab-in-the-field games to measure women’s empowerment (baseline, midline and endline) and risk attitudes (midline and endline only). As is standard in the behavioral economics literature, respondents can earn (small) amounts of money dependent on their decisions. This has been shown to improve accuracy of responses. The research team has extensive experience in conducting similar games in Kenya, Tanzania, Nigeria, and elsewhere. All payments are made directly to the respondent through M-PESA.

### Data management and analysis

Trained team leaders are situated in the field to supervise real time data collection. Data quality is ensured by regular spot checks and sit-ins to approximately 5–10% of each fieldworker’s daily work to verify authenticity of the data collected. Data collection is done electronically using tablets, with spot checks for quality control. The field supervisors certify the quality of the data through editing the data before they are transferred to the database. Once the data collection is completed and synchronized in a centrally located database, all inconsistencies are resolved prior to data analysis. Data quality is checked by a qualified data management team within the affiliated research organizations. An automated routine to check on data completeness, correctness, and consistency runs on 100% of the collected data. A discrepancy report is generated to help in following upon any inconsistencies, errors or missing data with the responsible interviewer. Similarly, the quality of qualitative data will be ensured through recruitment and training of qualified field interviewers with experience in qualitative data collection. A qualified transcriber will transcribe the interviews verbatim and double coding of about 10% of the transcripts will also be done to ensure consistency in the application of the codes. Access to data is granted for all research teams in respective research organizations. Data auditing is carried out by the research team on a progressive basis on weekly basis throughout the study duration.

The final analysis will investigate the extent of contamination (e.g., whether participants who were assigned to the control group nevertheless participated in the intervention) and appropriately account for it. The final will any risks of contamination, e.g., it will exclude those participants who are randomized to the intervention but do not adhere to the intervention.

Quantitative data will be analyzed using Stata version 14 statistical software. The first set of analysis will consist of descriptive statistics and will summarize and compare using measures of central tendency and dispersion (mean (SD), range and median). This will allow us to examine participants’ characteristics across the different sub-groups. We will first check whether the outcomes and covariates in the control group and treatment group are comparable at baseline using *t* test adjusted for clustering at the village unit for continuous variables and cluster-adjusted chi-square for binary variables. The second set of analysis will consist of assessing the causal effect of the *i*-PUSH intervention via an analysis of covariance (ANCOVA) based on intention-to-treat (ITT) analysis (all respondents who are randomized will be included in the statistical analysis and will be analyzed according to the group they were initially/originally assigned). To account for clustering of data at the village level, multi-level mixed models will be used. In addition, a generalized linear mixed model for repeated measures with random components will be used.

Based on our research questions, we will explore several additional econometric models. We will explore the impact of the *i*-PUSH program on health outcomes (healthcare utilization, health status, percentage of children who are fully immunized, etc.) and financial outcomes (percentage of women using NHIF/MTIBA to access services, percentage of people enrolled in NHIF through M-TIBA, percentage of people saving through M-TIBA, total amount saved on M-TIBA, etc.) via an analysis of covariance (ANCOVA) based on ITT analysis. Since we will have several rounds of data collection on the same respondents, we will combine all rounds of data collection (baseline and follow-up waves), and we will run an ANCOVA econometric model on a pooled panel data. More specifically, in the econometric model below, we will regress the follow-up measurement of the outcome variable (dependent variable) on the following covariates: baseline measurement (pretest measurement) and treatment group while controlling for the baseline socio-demographic characteristics such as maternal age, education, socioeconomic status (wealth quintile), crowding (persons per room), occupation, etc. The considered model is thus:

1 $$ {Y}_{ij}={\alpha}_0+{\alpha}_1{X}_{ij0}+{\alpha}_2{\mathrm{Treament}}_{ij}+{e}_{ij t}, $$

with *Y*_*ij*_, *X*_*ij*0_ the posttest and pretest/baseline measures of the outcome for the *ith* respondent from the jth cluster, respectively. Treament_*ij*_ is a dummy for being assigned to the treatment group. It takes one if the respondent belongs to the intervention cluster and zero if control. *α*_2_ is the ITT effect or the impact of the *i*-PUSH program. We will use an OLS model with standard errors clustered at the level of the community units.

In addition to pretest measures, all other baseline covariates such as age, education, socioeconomic status (wealth quintile), crowding (persons per room), and occupation, will also be included in Equation (1). Thus, in Equation (1), the treatment effect of the *i*-PSUH program *α*_2_ assesses the treatment difference on post-treatment outcome adjusted for baseline.

We will assess whether the *i*-PUSH will significantly contribute in empowering women. For self-reported surveys on women empowerment, we will construct a total score of women’s empowerment which is based on a factor analysis of the various domains on which the woman indicates that she has sole, joint, or no decision-making power within the household (for each domain, the decision-making binary indicator will be equal to 1 if the woman respondent makes the decision alone or jointly with her partner and 0 otherwise). This total score will be used as the dependent variable, and Equation (1) will be used to estimate the impact of *i*-PUSH program on women’s empowerment. Furthermore, we will follow Almås et al. (2018) to construct an alternative measure of women’s empowerment based on the decision in the empowerment game. This dependent variable will be the willingness-to-pay which is the share that the woman is willing to pay in order to receive the amount herself in private rather than giving a (higher) amount to her husband. Interim data analyses (e.g., for policy briefs) are done using baseline and weekly diaries data to inform the context and implementation of the research or *i*-PUSH program.

### Risks and measures to minimize them

As the information collected will be on health service delivery, financial, and insurance information, we do not anticipate any risks to participants. Nevertheless, we will aim to minimize any potential risks by being as forthcoming as possible on the project description and the ethical process. In the case of minimal discomfort as a result of personal and sensitive questions, the research team will endeavor to ensure that the participant is given ample time to compose themselves and reassure them of confidentiality and ability to stop the interview if they are not able to continue with the interview. Ancillary and post-trial care may not be indicated as this is a community trial that does not involve any significant harm to the participating households.

### Dissemination of findings

Scientific dissemination through peer-reviewed publications and implementing partners’ dissemination through quarterly updates (e.g., meetings, presentations and brainstorm sessions) to county/sub-county officials and community representatives will ensure that findings are shared on a regular basis. These activities allow all parties to benefit from each other’s insights and interpretations. Annual PharmAccess strategy meetings and AMREF international research meetings facilitate sharing knowledge with their respective country offices. Moreover, APHRC has good links with Kenyan policy makers in the field of health, and PAF is well connected to national and county-level government officials, the NHIF, as well as many local healthcare providers. Finally, the linkages between the research group, PAF and the Dutch Ministry of Foreign Affairs with respect to health financing ensure regular sharing of findings at the Dutch national policy level. The close connection of the AIGHD with the Joep Lange Institute and the Amsterdam Technology and Health Institute (ATHI) facilitates sharing of findings with parties active in social technologies (start-ups working on the interface of digital technologies and health, mobile payment platforms).

### Ethical considerations

The protocol was reviewed and approved by APHRC’s internal ethical review committee and AMREF’s accredited ethical review board. Ethical clearance was obtained on 21 April 2020 with number P679-2019. Research permits were obtained from the National Commission for Science, Technology and Innovation (NACOSTI) agency of Kenya. Informed consent from study participants was obtained upon detail orientation on project information, the purpose of the research study, possible risks, and benefits. The model consent form is provided in a separate file. All interviews are conducted in private and no identifying information is included in any data or reports; all data is anonymized to protect the identity of respondents. The participants are given a unique code and all identification data of participants shall be shredded/destroyed after analysis has been completed. Further ethical approval will be sought for any modifications to the protocol which may impact on the conduct of the study.

## Discussions

Despite various international efforts to improve maternal and child health globally, several LMICs, especially those in sub-Saharan Africa (SSA), are struggling with low rates of maternal and child survival. Improving access to health care throughout pregnancy, childbirth, and during childhood is key in improving maternal and child health. Experience over the past decade has shown that building capacities of individuals, families, and communities to ensure appropriate self-care, prevention and care-seeking behavior improves maternal and child health outcomes [5]. However, this is more difficult to achieve in poor populations who have worse health outcomes than the non-poor. Barriers such as costs of care, lack of information and cultural beliefs impede access to health care among poor communities.

Since its independence in 1963, the government of Kenya has initiated policy, reforms, and strategies towards UHC for all, including those in vulnerable situations such as low-income mothers and children. In 1998, the NHIF act was amended to enhance coverage among the poor, accelerate coverage of the informal sector, and enhance the benefit package [10, 11]. The most recent reform along the same line was the introduction of free maternal health services in 2013 that included abolition of user fees at primary health care facilities. Despite these positive steps, Kenya’s implementation of UHC has been riddled with myriads of challenges including poor quality of care, low utilization, and catastrophic spending by households, especially the poor and other vulnerable groups [11, 12]. Overall, health insurance coverage in Kenya has only increased from 8 to 20% between 2009 and 2014, and those from wealthy households were 12 times more likely to have insurance compared to those in poor households. Similarly, those in informal employment and rural settings were less likely to be insured [4].

The *i*-PUSH program thus has been initiated to accelerate the expansion of health insurance coverage among the low-income population, WRA and their family members, using innovative digital tools. This research investigates the reasons for low access to health services, in particular related to maternal and child services, where the problems are persistent. It also investigates in-depth to what extent costs of services hinder access, and whether expanding UHC through the *i*-PUSH program is an effective strategy to increase access and financial protection. Information generated from this study will be instrumental in improved implementation of policies supporting the roll-out of UHC. This research will also provide valuable information on UHC policies for the academic community, in particular, because we use high-frequency data (diaries) and analyze digital technologies that can support UHC.

To achieve these objectives, the study assumes the following: (i) no contamination across treatment and control groups; this is enhanced by a focus on villages and CHVs across villages, instead of individual women; (ii) the county (sub-county), AMREF, and PAF will stick to the randomization plan that was independently developed by the research team but in agreement with stakeholders, and a memorandum of understanding (MoU) was signed among the partners; and (iii) the government does not unexpectedly and drastically alter its UHC policy plans in Kakamega (i.e., the government does not suddenly decide to offer free public care in Kakamega County, because that will take away many of the benefits of *i*-PUSH). Although there is little the research team can do in that case, this is the reason a flexible, high-frequency diaries design instead of standard (less flexible) RCT design is chosen. That is, there are more data points and if policies change half way, it is possible to actually capture this in the data. For example, this has enabled the research team to adjust data collection methods halfway the study period to assess the impact of the COVID-19 pandemic.

This project is not free from limitations. One of the main limitations of the study is related to the exclusion of the following components in the impact evaluation, though they are an integral part of *i*-PUSH program. These include four components:

(i) Capacity-building at the regional level: *i*-PUSH invests in improving the capacity of community-based organizations to increase community-wide dialog on RMNCH,
(ii) Health facility quality upgrades: the improved capacity of healthcare providers (implemented through SafeCare) to deliver RMNCH services is expected to lead to improved standards of care, improved quality of services and enhanced client satisfaction.
(iii) M-JALI household registration tool: AMREF has developed a digital tool to increase the capacity of CHVs to keep a census of the households in their target area, and collect household survey data on a rolling basis that can be used for the systematic reporting of community-level data, thereby potentially enhancing health-related decision-making and resource allocation at all policy levels.
(iv) M-TIBA digital health platform: PAF aims to increase the capacity of healthcare workers on learning, data capturing and reporting through the M-TIBA platform that allows among others for digital recording of health visits, diagnoses, treatments, and services as well as payments/billing between patients, NHIF, Ministry of Health, and providers.

These components are left out of the evaluation because our study focuses on the demand-side (target population), and the described components are implemented either at the community-level or at the facility-level.

Furthermore, there may be a potential for Hawthorne effects because of the weekly visits. However, since both the treatment and the control group are included in the weekly diaries data collection, we expect the selectivity of this effect to remain limited.

## Conclusions

This study aims to evaluate the impact of *i*-PUSH program on maternal and child health care utilization, women’s health including their knowledge, behavior, and uptake of respective services, as well as women’s empowerment and financial protection. It also aims to evaluate the impact of the LEAP training tool on empowering and enhancing CHVs’ health literacy and to evaluate the impact of the M-TIBA health wallet on savings for health and health insurance uptake. The findings will inform stakeholders to formulate better strategies to ensure access to UHC in general and for those highly vulnerable segments of the population in particular. The findings of this research will provide valuable information on UHC policies for the academic community, policy-makers, and other stakeholders to support the achievement of SDGs.

## Trial status

Protocol was registered in NIH - ClinicalTrials.org with registration number: AfricanPHRC; Trial ID: NCT04068571 dated on 28 August 2019 (https://clinicaltrials.gov/ct2/show/NCT04068571). Protocol amendment number: 01. It was also registered in The American Economic Association's registry for randomized controlled trials (Trial ID: AEARCTR-0006089) dated on 26 June 2020 (https://www.socialscienceregistry.org/trials/6089). Recruitment began in November 2019 and will continue until June 2021.

## Data Availability

Data will be stored securely at the APHRC and Vrije Universiteit data repositories and available upon reasonable request after publication.

## Appendix

**Table 2:** Modules covered in the study, unit of responses and respondents

Modules	Unit of response	Respondent
1. Cover page	Household	Enumerator
2. Household roster	All household members	Head/most informed member
3. Socio-demographics	All household members	Head/most informed member
4. Education	All household members > 5 years	Head/most informed member
5. Health outcomes	All household members	Head/most informed member
6. Health care utilization	All household members	Head/most informed member
7. Food consumption	Child < 4 years	Head/most informed member
8. Housing and assets	Household	Head/most informed member
9. Employment and income	All household members > 12 years	Head/most informed member
10. Participation in diaries	All adults or emancipated minor	Individual
11. Women’s empowerment	Female adult or emancipated minor	Individual
12. Preferred clinics and mobile money	All adults or emancipated minor	Individual
13. Mental health	All adults or emancipated minor	Individual
14. Women’s health	Female adult or emancipated minor	Individual
15. Savings groups and cooperatives	All adults or emancipated minor	Individual
16. Financial instruments	All adults or emancipated minor	Individual
17. Health knowledge	Female adult or emancipated minor	Individual
18. COVID-19 module	All adults or emancipated minor	Individual

## Abbreviations

AIGHD: Amsterdam Institute for Global Health and Development
AMREF: African Medical and Research Foundation
ANC: Antenatal care
APHRC: African Population and Health Research Center
CHV: Community health volunteer
FGD: Focus group discussion
ICC: Intracluster correlation
IDI: In-depth interview
ITT: Intention-to-treat
*i*-PUSH: Innovative Partnership for Universal Sustainable Healthcare
LMICs: Low and middle-income countries
KDHS: Kenya Demographic and Health Survey
NACOSTI: Memorandum of understanding
NACOSTI: National Commission for Science, Technology and Innovation
NHIF: National Health Insurance Fund
PAF: PharmAccess Foundation
RCT: Cluster randomized controlled trial
RMNCH: Reproductive and Maternal and Child Health
SDG: Sustainable Development Goal
SSA: Sub-Saharan African
WRA: Women of reproductive age
UHC: Universal Health Coverage

## Footnotes

1 PAF is currently working on new approaches to differentiate the size of the co-payment by socioeconomic status, which could be more effective in targeting subsidized health insurance to those who cannot afford the full premium, while at the same time, introducing new incentives to save for co-payments for those who can afford them.
